# Determinants of community health workers effectiveness for delivery of maternal and child health in Sub Saharan Africa: A Systematic review protocol

**DOI:** 10.1371/journal.pone.0271528

**Published:** 2022-07-19

**Authors:** Akalewold T. Gebremeskel, Olumuyiwa Omonaiye, Sanni Yaya

**Affiliations:** 1 Faculty of Health Sciences, University of Ottawa, Ontario, Canada; 2 School of International Development and Global Studies, University of Ottawa, Ottawa, Ontario, Canada; 3 Centre for Quality and Patient Safety Research, School of Nursing and Midwifery, Melbourne Burwood Campus, Burwood, Australia; 4 Centre for Nursing and Midwifery Research, James Cook University, Townsville, Queensland, Australia; 5 University of Parakou, Faculty of Medicine, Parakou, Benin; 6 The George Institute for Global Health, Imperial College London, London, United Kingdom; Flinders University, AUSTRALIA

## Abstract

**Background:**

Countries in sub-Sahara African continue to have the highest maternal and under- five child death occurrences in the world and this has become a key health challenge in the region and persists as global public health agenda. Although Community Health Workers (CHWs) are increasingly being acknowledged as crucial members of the healthcare workforce in reducing health disparity, evidence is limited on perspective of community health workers. The objective of this protocol is to outline the methodological process of a systematic review that will gather qualitative data to examine determinants of community health workers effectiveness for delivery of maternal and child health in Sub Saharan Africa. Synthesizing the perspectives of community health workers’ perceived experience is crucial to inform decision makers, policy makers, and practitioners to address barriers to and scaleup facilitators of CHWs program to ensure maternal and child health equity and a resilience community health system.

**Methods:**

The protocol has been registered in the PROSPERO (CRD42020206874). We will systematically conduct a literature search from inception in MEDLINE complete, EMBASE, CINAHL complete and Global Health for relevant studies. Eligible studies will be reports of original research, peer reviewed articles having a qualitative component (i.e., qualitative, mixed, or multi-method studies) on empowerment of CHWs associated with maternal and child health in the sub-Saharan Africa. Eligibility will be restricted to studies published in English. Two reviewers will independently screen all included abstracts and full-text articles. The primary outcome will be CHWs’ perceived barriers to and facilitators of effectiveness of community health workers in maternal and child health in sub-Saharan Africa. Study methodological quality (or bias) will be appraised using appropriate tools. Narrative analysis will be conducted, and narrative summary of findings will be presented. We will use the ‘best fit’ framework method as a systematic approach to analyzing the qualitative data.

**Discussion:**

This study will systematically and comprehensively search literature and integrate evidence on perceived barriers to and facilitators of effectiveness of community health workers led maternal and child health program in sub-Saharan Africa. Our findings will inform policy and practice on maternal and child health equity and a resilient communities health system. The resulting manuscript will be disseminated in a peer-reviewed journal and at international and national conferences.

## Background

Despite the reduction of inequality in the last two decades in the area of Maternal and Child Health(MCH) outcome, the health system in sub-Saharan Africa, particularly the community based health system is still not equitable and resilient enough to respond to existing public health issues including MCH [1, 2]. Although Community Health Workers (CHWs) are increasingly being acknowledged as crucial members of the healthcare workforce in reducing health disparity, the countries in sub-Saharan Africa continue to have the highest maternal and under-five child death occurrences in the world. Hence, this has become a key health challenge in the region and persists as a global public health agenda for the new Sustainable Development Goals [3, 4]. Multiple evidence suggests that scaling up community health program and empowerment of CHWs can lead to better health outcomes [5, 6]. Over the past several years, increased attention has been given to CHWs led programs. However, challenges remain regarding ensuring adequate health systems support for CHWs [5].

World Health Organization (WHO) defined health system as all organizations, people and actions whose primary intent is to promote, restore, or maintain health [7]. Health manpower is among the essential component of a resilient health system [8]. However, in sub-Saharan Africa the health workforce remains four times below the recommended WHO standard [9] to ensure health equity. Health equity is the state in which all people are able to reach their full health potential and receive high quality care that is fair and appropriate from each person’s perspective, no matter where they live, who they are or what they have [10]. Health system resilience can be defined as the capacity of health actors, institutions, and populations to prepare for and effectively respond to crises; maintain core functions when a crisis hits; and, informed by lessons learned during the crisis, reorganize if conditions require it [11]. Health coverage, quality of care and continuum of care are integral part of health equity and a resilient health system.

MCH inequality persist as an important agenda of the new Sustainable Development Goal [12]. Sustainable Development Goal 3 was aimed at reducing maternal mortality to less than 70 per 100,000 live births; reduce newborn mortality to at least as low as 12 per 1,000 live births in every country; and reducing under-five mortality to at least as low as 25 per 1,000 live births in every country [13, 14]. The Millennium Development Goal 5, which aimed to reduce maternal mortality rate by three-quarters, was not achieved in sub-Saharan Africa [13, 15]. In sub-Saharan Africa, a larger proportion of women give birth without any skilled attendants [13]. In rural areas, only 56 per cent of births were attended by skilled health personnel, compared with 87 per cent in urban areas [13]. In low and middle income countries, many of which are in sub-Sahara Africa, the coverage of institutional delivery is as low as 50 per cent but accounts for 99 percent of global maternal mortality [2]. In addition, about 80% of morbidity and death in children under the age of five occur before reaching health institution and health care providers [11]. This makes sub-Sahara Africa the riskiest region for maternal and under- five child death occurrences in the world. Understandably, this has become a key health challenge in the region.

In sub-Sahara Africa the unacceptable health outcomes including MCH are associated with health system challenge [16]. There is a shortage of 3.7 million healthcare workers to respond to the continued health crises [17]. For example, the region accounts 25% of the world’s disease burden, however spends less than 1% from Gross Domestic Product on health and has only 3% of the world’s health manpower [18]. Particularly, the empowerment and availability of CHWs is not comparable to the demand, which poses a real challenge to the already fragile health system. Thereby, reducing the capacity of the health system to respond to the exceptional high maternal and child mortality rates in the region. The situation is worse in rural and remote areas where the provision of services is difficult because of limited health budgets and scattered populations living in isolated villages or islands [19]. We argue that context based effective CHWs empowerment could be a useful strategy to address the health workers challenge to ensure MCH equity and a resilient health system. Empowerment has become a dynamic mainstream action-oriented concept with a focus on removal of barriers and enhancement effective development of policy and practice [20, 21].

Community Health Workers led programs have contributed significantly to improving public health outcomes in sub-Saharan Africa [5, 22]. The WHO has noted CHWs, as a key resource to providing basic health services for underserved areas because they help to fill the shortage of primary health service provider at the community level. Since 1978, Alma Ata’s call there have been multiple efforts towards building community health [17, 18] to play major role in undeserved countries including countries in sub-Saharan Africa [19]. According to the WHO, CHWs are members of the communities where they work, who should be selected by the communities, be supported by the health system and have shorter training than professional workers [15, 16]. They are more accessible and they provide the platform that ensure health equity [23]. CHWs provide non-heretical community-based services. This is because they are well positioned in connecting grass-root communities to the public health system and thereby making the health system more people-centered to promote, prevent, detect and respond to primary health needs including MCH [24]. The promising outcomes of CHWs in providing equitable MCH services is noticeable in many countries such as Brazil, Bangladesh, and Nepal which have effectively executed CHWs led programmes [5, 18, 22–24]. However, while community health programs are conceptually and operationally related to specific community setting, there is limited evidence on context-based community health system building. Health system approach aligned to or based on the context is crucial to plan, implement and sustain a resilient community health system.

Although there is an increasing body of literature on effectiveness (What works) of empowerment of CHWs program from the intervention outputs and outcome measures, and health policy and beneficiary community, there is limited evidence on the process of how CHWs programs work (how it work)from the lived or practical experience and perspective of CHWs [5]. Existing studies have focused on effectiveness of CHWs program intervention result and target from the perspective of beneficiary community [25–28]. Furthermore, some of these studies are in the context of global to low- and middle-income countries [29, 30] and they are not specifically in the context of sub-Sahara Africa. Additionally, these studies have reported the barriers of effectiveness of the empowerment of CHWs based on quantitative measurement such as output and outcome measures [17, 25].

We argue that evidence based on result measures cannot tell how the empowerment strategy facilitated or disrupted the power of CHWs on their work environment, ensuring MCH equity and a resilient community health system. Hence, it would be difficult to identify and address where and when CHWs feel powerlessness and frustrated about how organizational and relational arrangements hindered them from achieving the desired impact, particularly in reducing MCH inequality and building a resilient community health system. Also, measurement of output and outcome indicator metrics do not fully capture process and quality of care and continuum of care and are limited to measuring contact with a health provider. Measuring outcomes alone does not provide insight into the causal link between the process and outcome. Hence it is insufficient in capturing whether people receive quality care or how it works [2, 31]. For example, the work by Stiglitz-Sen-Fitoussi on going “Beyond GDP”, indicates that a singular focus on outcomes is insufficient to show “how the pie was sliced” [32].

According to the WHO there is a research gap in understanding how to ensure the sustainability of CHWs program, previous research experience on the role of community-based health workers represents a mix of varying degrees of quality [33]. The existing reviews highlight a lack of robust evidence on contextual factors from the perspective of community health workers. Thus, to the best of our knowledge there is no systematic review that has provided in-depth insight into the individual and contextual barriers to and enablers of the effectiveness of CHWs from the perspective of front line CHWs. In addition, to the best of our knowledge no systematic review has been performed to unravel the contextual factors like how, for whom and under what circumstances the CHWs’ programs work.

The objective of this protocol is to outline the methodological process of a systematic review that will gather qualitative data to examine determinants of community health workers effectiveness for delivery of maternal and child health in Sub Saharan Africa.

This systematic review will be guided by the following question: What are the CHWs’ perceived barriers to and facilitators of effectiveness of community health workers to ensure MCH equity and a resilient community health system in sub-Saharan Africa?

## Methods

### Protocol registration and reporting

The registration number of this protocol in PROSPERO is CRD42020206874. The Preferred Reporting Items for Systematic Reviews and Meta-Analyses Protocols (PRISMA-P) statement [34] guided the reporting of this protocol (see checklist in S1 File). The proposed systematic review will be conducted according to the Cochrane Collaboration Handbook of Systematic Reviews [35] and reported in accordance with the reporting guidance provided in Enhancing Transparency in Reporting the Synthesis of Qualitative Research (ENTREQ) statement [36] and the Preferred Reporting Items for Systematic Reviews and Meta-analyses (PRISMA) statement [34].

### Search methods for identification of studies

#### Electronic searches

Four major electronic databases will be searched: MEDLINE (Ovid), EMBASE, CINAHL and Global Health for relevant peer-reviewed articles published between 2000 and 2021. The search strategies are designed to access published materials in three stages: (i) A limited search of Ovid Medline to identify relevant keywords contained in the title, abstract and subject descriptors; (ii) Terms identified in this way, and the synonyms used by Ovid Medline, EMBASE, CINAHL, and Global Health are used in an extensive search of the literature; (iii) We will perform hand-searching of the reference lists of the review eligible full-text articles to identify and include more relevant articles. The searches will be designed and conducted by the review team which includes three experienced public health researchers, in collaboration with a Health Sciences librarian. They will help in optimizing the retrieval of relevant citations: planning, searching, citation management, source selection, and bias assessment. A comprehensive search will be conducted involving a broad range of MeSH terms and keywords related to MCH services and CHWs. A draft search strategy is provided in S2 File.

#### Study inclusion criteria

*Population*. We will include studies involving CHWs engaged in community setting under public health system with some level of secondary education; subsequent formal training from a recognized institution training **and salaried.** For this study, we will use the definition of CHWs provided by the WHO and American Public Health Association [37, 38].

#### Intervention/ exposure context

*Intervention/ exposure context*. Eligible studies will involve different empowerment programs designed to empower CHWs work associated with MCH services. The services include MCH promotion, follow up and linkage, family planning, antenatal, delivery, post natal care, breastfeeding, immunization/ vaccination, and newborn services for mothers and under five children in public health system in sub-Saharan Africa.

*>Comparison or control group.* No comparison group for this study

*Outcomes of interest*. The primary outcome will be CHWs’ perceived barriers to and facilitators of effectiveness of CHWs to ensure MCH equity and a resilient community health system in sub-Saharan Africa.

*Setting*. We will include studies conducted on CHWs empowerment/program experience based in community setting under public health systems in countries of sub-Saharan Africa, which includes countries in Eastern, Central, Western and Southern regions of African continents.

*Study design*. Eligible studies will be reports of original research, peer reviewed articles, dissertations, and gray literature (e.g., reports) having a qualitative component (i.e., qualitative, mixed, or multi-method studies) conducted in sub-Saharan Africa with focus on empowerment of CHWs. The study will include the lived experience of CHWs (on job training or practical experience), experience after the empowerment intervention. The eligible studies must be published in English language between January 2000 to September 2021. This period was selected because the Millennium Development Goal was implemented during this period. The current Sustainable Development Goals have been implemented since 2016. Both development programs placed emphasis on MCH. Hence, 2000 to 2021 represents the period where substantial international resources were channeled towards alleviating the poor state of MCH in developing countries.

#### Exclusion criteria

We will exclude studies based on the following criteria: Studies on CHWs reporting on performance of CHWs as an output and outcome from the perspective of beneficiary community will not be considered; Studies based on CHWs while in school or during training or workshop time; studies on quantitative methods; studies based on volunteers/unpaid, nurses, midwiferies; studies on empowerment not related to family planning, pregnancy and childbirth related, under five children and the like; studies on empowerment of CHWs on environmental health, and sanitation and the like; studies not reporting on CHWs’ perspective on barriers to and facilitators of effectiveness of empowerment of CHWs; studies conducted before January 2000 and published in languages other than English language; and studies based on conference abstracts and commentaries and out of public health settings in sub-Saharan Africa, will not be included.

### Data collection and analysis

#### Selection of studies

The articles retrieved from searches in each database will be uploaded into Covidence. Two authors (AG&OO) will independently screen articles in Covidence. This process will involve screening titles and abstracts. Afterwards, full text articles will be screened against the predefined eligibility criteria. To document the selection process, the PRISMA (Preferred Reporting Items for Systematic Review and Meta-Analyses) flowchart will be used [39].

#### Data extraction and management

Once, full text data screening has been completed, two authors will independently extract data from articles meeting the inclusion criteria. If there are disagreements, the third author will serve as an arbiter. A standardized data extraction form from the Cochrane library will be adapted for this the review [35, 40]. From each article, information such as the (i) authors and publication year, study setting, and study aim or hypothesis; (ii) CHWs empowerment/ intervention details (setting, content, format, duration); (iii) sample characteristics, design and data collection methods, outcome measures; (iv) study findings; (v) CHWs’ perceived barrier to and facilitators of effectiveness of empowerment of CHWs engagement in MCH program and building a resilient community will be extracted. Primary authors of included studies will be contacted if essential information is missing or not clear.

#### Certainty of evidence

To appraise and summarize confidence in key findings, the GRADE-CERQual (“Confidence in the Evidence from Reviews of Qualitative research”) approach will be applied by two authors independently. GRADE-CERQual summary of qualitative findings tables will be used to present results [41].

### Assessment of risk of bias in included studies

#### Appraisal of study quality

Methodological rigor in this review will be conducted by having two (AG&OO) reviewers independently. To evaluate the qualitative, mixed, or multi-method studies, the reviewers will use the appropriate Critical Appraisal Skills Programme checklists. The domains of the CASP checklists will help to assess the credibility of the findings and the rigor of the studies [42]. The use of these questions will aid in guiding the reviewers when critically reading the articles. The tool has ten questions that each focus on a different methodological aspect of a qualitative study. Then reviewers will determine the quality of the questions using three weighting categories: if “Yes and high” quality then the question will weight “1”; if “Yes but moderate (weight below)” quality then the question will weight 0.5; or if “below moderate and No” quality the question will weight “0”. Then, to determine the quality of the study the total will be added out of ten. Accordingly, included studies will be assigned an overall score of ‘high’ (9–10), ‘moderate’ (7.5–9) or ‘low’ (less than 7.5) overall quality. Studies will not be excluded based on the quality of the reporting assessment. Rather the results of the appraisal will be used to inform data interpretation. Differences in the quality assessment will be resolved by discussion among all the authors. The discrepancies will be resolved by discussion with a third reviewer (SY).

### Data synthesis

We will report the results according to the Cochrane Handbook of Systematic Reviews of Interventions [35]. A PRISMA flowchart will be used to present the process of study selection for both the overview and the systematic review.

Evidence tables of an overall description of the included studies will be provided. The evidence tables will comprise information such as study setting, country, study type, participant characteristics, review objective and outcome, MCH services type, participant (CHWs) age and sex.

A narrative synthesis will be conducted. This approach is used for synthesis of findings from multiple study designs that depend fundamentally on the use of words and text to summarize and explain the findings of the synthesis [43, 44]. Synthesis of data will be described in a narrative synthesis grouped by study type, participant characteristics, review objective and outcome. We will provide a narrative synthesis of the review results (CHWs’ perceived barriers to and facilitators of effectiveness of empowerment of community). We will use the ‘best fit’ framework method as a systematic approach to analyzing the qualitative data [45, 46]. Framework-based synthesis using the ‘best fit’ strategy is a highly pragmatic and useful approach for a range of policy related questions and understanding complex context [47]. Framework analysis is a five-stage process that includes familiarization with the data, identifying a thematic framework, indexing (applying the framework), charting and mapping, and interpretation [48]. We will determine the appropriate framework based on team discussions.

## Discussion

This protocol outlines the methodological process of a systematic review that will gather data to examine the determinants of CHWs effectiveness for equitable maternal and child health, and a resilient community health system in Sub Saharan Africa.

Such an evidence base has the potential to provide an opportunity to better plan and implement MCH. Hence, this review will be of value to decision makers, policy makers, practitioners, and members of the community with interest in supporting the MCH equity and a resilient community health system. Additionally, this review will contribute to the campaign for effective empowerment of CHWs. However, some limitations are anticipated when planning the methodology for this systematic review. Because of the exclusion of studies not published in English language, important data may be missed. This may result in publication bias. The resulting manuscript will be disseminated in a peer-reviewed journal and at international and national conferences.

## Supporting information

S1 File Preferred Reporting Items for Systematic Review and Meta-analysis Protocols (PRISMA-P). (DOCX)

S2 File Search terms. (DOCX)

S1 Data (PDF)

## Abbreviations

CASP: Critical Appraisal Skills Programme
CHWs: Community Health Workers
MCH: Maternal and Child Health
PRISMA-P: Preferred Reporting Items for Systematic Review and Meta-analysis Protocols
PROSPERO: International Prospective Register of Systematic Reviews
WHO: World Health Organization
