# Supplementary material for: Determinants of community health workers effectiveness for delivery of maternal and child health in Sub Saharan Africa: A Systematic review protocol
Source: PLoS One. 2022 Jul 19;17(7):e0271528. doi: 10.1371/journal.pone.0271528 (PMC9295951; doi:10.1371/journal.pone.0271528)
Supplement: S2 File — (DOCX) [file pone.0271528.s002.docx]

Additional File 2. Search Terms

Search Terms: Medline (OVID)

**Set 1: Intervention**

1. *MeSH*

Maternal and child adj2 health adj2 (service* or Program*) or community health*

1. *Keyword*

Maternal health / or Under-five child health / exp contraceptive/ or exp antenatal care/ or exp delivery/ or exp postnatal care/ or exp breastfeeding/ or nutrition/ or exp immunization/ or exp vaccination / or exp newborn services

*C) Keyword*

Empowerment and financial or in kind resources or career development opportunity or training or motivation or certificate

**Set 2: Population**

1. *MeSH*

exp health auxiliary/ or Community health worker*or CHWs/ or Lay health worker*

1. *Keyword*

Sub-Saharan Africa and all names of each country and the regions (Eastern, Central, Western and Southern Africa

**Set 3: Search Alone**

*A) MeSH*

Women/

*B) Keyword*

Wom?n or or female*

**Set 4: Study Design**

Qualitative Research/ or Interview/ or Mixed research or multi level research

**Set 5: Outcomes**

CHWs perception and barriers/facilitators and effectiveness

**Search Summary:** Set 1 (A or B or C) and Set 2 (A or B ) and Set 3 (A or B) and Set 4 and Set 5
