# Supplementary material for: Determinants of community health workers effectiveness for delivery of maternal and child health in Sub Saharan Africa: A Systematic review protocol
Source: PLoS One. 2022 Jul 19;17(7):e0271528. doi: 10.1371/journal.pone.0271528 (PMC9295951; doi:10.1371/journal.pone.0271528)
Supplement: S1 Data — (PDF) [file pone.0271528.s003.pdf]

NHS
National Institute for Health Research

UNIVERSITY of York
Centre for Reviews and Dissemination

Systematic review

This record cannot be edited because it has been marked as out of scope

1. * Review title. [2 changes]

Give the title of the review in English

Determinants of community health workers effectiveness for delivery of maternal and child health in Sub Saharan Africa: A Systematic review protocol

2. Original language title.

For reviews in languages other than English, give the title in the original language. This will be displayed with the English language title.

3. * Anticipated or actual start date.

Give the date the systematic review started or is expected to start.

15/06/2020

4. * Anticipated completion date. [1 change]

Give the date by which the review is expected to be completed.

04/02/2024

5. * Stage of review at time of this submission. [1 change]

This field uses answers to initial screening questions. It cannot be edited until after registration.

Tick the boxes to show which review tasks have been started and which have been completed.

Update this field each time any amendments are made to a published record.

The review has not yet started: No

Review stage	Started	Completed
Preliminary searches	Yes	Yes
Piloting of the study selection process	Yes	Yes
Formal screening of search results against eligibility criteria	Yes	No
Data extraction	No	No
Risk of bias (quality) assessment	No	No
Data analysis	No	No

Provide any other relevant information about the stage of the review here.

6. * Named contact.

The named contact is the guarantor for the accuracy of the information in the register record. This may be any member of the review team.

Sanni Yaya

Email salutation (e.g. "Dr Smith" or "Joanne") for correspondence:

Sanni Yaya, PhD, Hononary Professorial Fellow

7. * Named contact email.

Give the electronic email address of the named contact.

SYaya@georgeinstitute.org.uk

8. Named contact address

Give the full institutional/organisational postal address for the named contact.

The George Institute for Global HealthUniversity of Oxford,

Faculty of Medicine, Imperial College London, London, SW7 2AZ, United Kingdom

9. Named contact phone number.

Give the telephone number for the named contact, including international dialling code.

44 1865 617200

10. * Organisational affiliation of the review.

Full title of the organisational affiliations for this review and website address if available. This field may be completed as 'None' if the review is not affiliated to any organisation.

The University of Oxford

Organisation web address:

The George Institute for Global HealthUniversity of Oxford,

Faculty of Medicine, Imperial College London, London, SW7 2AZ, United Kingdom

11. * Review team members and their organisational affiliations.

Give the personal details and the organisational affiliations of each member of the review team. Affiliation refers to groups or organisations to which review team members belong. **NOTE: email and country now MUST be entered for each person, unless you are amending a published record.**

Dr Sanni Yaya. The University of Oxford
Mr Akalewold Gebremeskel. School of International Development and Global Studies, University of Ottawa, Ottawa, Ontario, Canada
Dr Olumuyiwa Omonaiye. Centre for Quality and Patient Safety Research, School of Nursing and Midwifery, Melbourne Burwood Campus, Australia

12. * Funding sources/sponsors.

Details of the individuals, organizations, groups, companies or other legal entities who have funded or sponsored the review.

Not Applicable

Grant number(s)

State the funder, grant or award number and the date of award

13. * Conflicts of interest.

List actual or perceived conflicts of interest (financial or academic).

None

None

14. Collaborators.

Give the name and affiliation of any individuals or organisations who are working on the review but who are not listed as review team members. **NOTE: email and country must be completed for each person, unless you are amending a published record.**

15. * Review question. [2 changes]

State the review question(s) clearly and precisely. It may be appropriate to break very broad questions down into a series of related more specific questions. Questions may be framed or refined using PI(E)COS or similar where relevant.

What are the CHWs' perceived barriers to and facilitators of effectiveness of community health workers to ensure MCH equity and a resilient community health system in sub-Saharan Africa?

16. * Searches. [2 changes]

State the sources that will be searched (e.g. Medline). Give the search dates, and any restrictions (e.g. language or publication date). Do NOT enter the full search strategy (it may be provided as a link or attachment below.)

We will systematically conduct a literature search from inception in MEDLINE complete, EMBASE, CINAHL complete and Global Health for relevant peer-reviewed articles CHWs engagement in MCH in SSA.

Research articles incorporating different methods (quantitative, qualitative and mixed methods) published

between January 2000 to September 2021 and in English language.

17. URL to search strategy.

Upload a file with your search strategy, or an example of a search strategy for a specific database, (including the keywords) in pdf or word format. In doing so you are consenting to the file being made publicly accessible. Or provide a URL or link to the strategy. Do NOT provide links to your search **results**.

Alternatively, upload your search strategy to CRD in pdf format. Please note that by doing so you are consenting to the file being made publicly accessible.

Yes I give permission for this file to be made publicly available

18. * Condition or domain being studied. [1 change]

Give a short description of the disease, condition or healthcare domain being studied in your systematic review.

We will include studies conducted on CHWs empowerment experience based in community setting under public health systems in countries of sub-Saharan Africa, which includes countries in Eastern, Central, Western and Southern regions of African continents.

19. * Participants/population. [1 change]

Specify the participants or populations being studied in the review. The preferred format includes details of both inclusion and exclusion criteria.

Type of participants/Population: We will include studies involving CHWs engaged in community setting under public health system with some level of secondary education; subsequent formal training from a recognized training institution; and salaried.

20. * Intervention(s), exposure(s). [1 change]

Give full and clear descriptions or definitions of the interventions or the exposures to be reviewed. The preferred format includes details of both inclusion and exclusion criteria.

Intervention/ Exposure context: Eligible studies will involve different empowerment programs designed to empower CHWs work associated with MCH services. The services include MCH promotion, follow up and linkage, family planning, antenatal, delivery, post natal care, breastfeeding, immunization/ vaccination, and newborn services for mothers and under five children in public health system in SSA.

21. * Comparator(s)/control.

Where relevant, give details of the alternatives against which the intervention/exposure will be compared (e.g. another intervention or a non-exposed control group). The preferred format includes details of both inclusion and exclusion criteria.

No comparison group for this study

22. * Types of study to be included. [1 change]

Give details of the study designs (e.g. RCT) that are eligible for inclusion in the review. The preferred format includes both inclusion and exclusion criteria. If there are no restrictions on the types of study, this should be stated.

Study design: Eligible studies will be reports of original research, peer reviewed articles, dissertations, and gray literature (e.g., reports) having a qualitative component (i.e., qualitative, mixed, or multi-method studies) conducted in sub-Saharan Africa with focus on empowerment of community health workers. The study will include the lived experience of CHWs (on job training or practical experience), experience after the empowerment intervention. The eligible studies must be published in English language between January 2000 to September 2021. This period was selected because the MDGs was implemented during this period. The current SDGs has been implemented since 2016. Both development programs placed emphasis on MCH. Hence, 2000 to 2021 represents the period where substantial international resources were channeled towards alleviating the poor state of MCH in developing countries.

23. Context. [[illegible] changes]

Give summary details of the setting or other relevant characteristics, which help define the inclusion or exclusion criteria.

Setting: We will include studies conducted on CHWs empowerment/program experience based in community setting under public health systems in countries of sub-Saharan Africa, which includes countries in Eastern, Central, Western and Southern regions of African continents.

24. * Main outcome(s). [[illegible] changes]

Give the pre-specified main (most important) outcomes of the review, including details of how the outcome is defined and measured and when these measurement are made, if these are part of the review inclusion criteria.

The primary outcome will be CHWs' perceived barriers to and facilitators of effectiveness of CHWs to ensure MCH equity and a resilient community health system in sub-Saharan Africa.

Measures of effect

Please specify the effect measure(s) for you main outcome(s) e.g. relative risks, odds ratios, risk difference, and/or 'number needed to treat.

Not applicable

25. * Additional outcome(s).

List the pre-specified additional outcomes of the review, with a similar level of detail to that required for main outcomes. Where there are no additional outcomes please state 'None' or 'Not applicable' as appropriate to the review

Not applicable

Measures of effect

Please specify the effect measure(s) for you additional outcome(s) e.g. relative risks, odds ratios, risk difference, and/or 'number needed to treat.

26. * Data extraction (selection and coding). [[illegible] changes]

Describe how studies will be selected for inclusion. State what data will be extracted or obtained. State how this will be done and recorded.

Selection of studies:

The articles retrieved from searches in each database will be uploaded into the Covidence article online

management system to be screened by two authors (AG&OO) within the Covidence database for their relevance and eligibility to the review. This will include title and abstract screening, followed by full-text screening against the eligibility criteria for studies deemed potentially eligible. The PRISMA (Preferred Reporting Items for Systematic Review and Meta-Analyses) flowchart will be used to document the selection process (39).

Data extraction and management

Once, full text data screening has been completed, two authors will independently extract data from articles meeting the inclusion criteria. If there are disagreements, the third author will serve as an arbiter. A standardized data extraction form from the Cochrane Library will be adapted for this the review(35)(40). From each article, information such as the (i) authors and publication year, study setting, and study aim or hypothesis; (ii) CHWs empowerment/ intervention details (setting, content, format, duration); (iii) sample characteristics, design and data collection methods, outcome measures; (iv) study findings; (v) CHWs' perceived barrier to and facilitators of effectiveness of empowerment of CHWs engagement in MCH program and building a resilient community will be extracted. Primary authors of included studies will be contacted if essential information is missing or not clear.

21. * Risk of bias (quality) assessment. [1 change]

State which characteristics of the studies will be assessed and/or any formal risk of bias/quality assessment tools that will be used.

Assessment of risk of bias in included studies

Appraisal of study quality

Methodological rigor in this review will be conducted by having two (AG&OO) independent reviewers critically appraising the methodological validity of the included studies. The reviewers will evaluate the qualitative, mixed, or multi-method studies using the appropriate Critical Appraisal Skills Programme checklists. The domains of the CASP checklists will help to assess the credibility of the findings and the rigor of the studies(42). The use of these questions will aid in guiding the reviewers when critically reading the articles. Studies will not be excluded or weighted based on the quality of the reporting assessment. The results of the appraisal will instead be used to inform data interpretation and help confirm the validity of review findings and conclusions. Differences in the quality assessment will be resolved by discussion among all the authors. The discrepancies will be resolved by discussion with a third reviewer (SY).

22. * Strategy for data synthesis. [1 change]

Describe the methods you plan to use to synthesise data. This **must not be generic text** but should be **specific to your review** and describe how the proposed approach will be applied to your data. If meta-analysis is planned, describe the models to be used, methods to explore statistical heterogeneity, and software package to be used.

Evidence tables of an overall description of the included studies, including data from each paper that provided details of study characteristics such as study setting, setting, country, study type, participant characteristics, review objective and outcome, MCH services type, participant (CHWs) age and sex will be used to build evidence tables for eligible studies to provide an overall description of included studies.

A narrative synthesis will be conducted, a method that is ideal for synthesizing evidence from a wide range of research questions and study designs with qualitative and mixed-method approaches, as the emphasis is on an interpretive synthesis of the narrative findings of research (43)(44). Synthesis of data will be described in a narrative synthesis grouped by study type, participant characteristics, review objective and outcome. We will provide a narrative synthesis of the review results (CHWs' perceived barriers to and facilitators of effectiveness of empowerment of community). We will use the 'best fit' framework method as a systematic approach to analyzing the qualitative data (45)(46). Framework-based synthesis using the 'best fit' strategy is a highly pragmatic and useful approach for a range of policy related questions and understanding complex context(47). Framework analysis is a five-stage process that includes familiarization with the data, identifying a thematic framework, indexing (applying the framework), charting and mapping, and interpretation (48). We will determine the appropriate framework based on team discussions.

29. * Analysis of subgroups or subsets.

State any planned investigation of 'subgroups'. Be clear and specific about which type of study or participant will be included in each group or covariate investigated. State the planned analytic approach.

Not applicable

30. * Type and method of review. [1 change]

Select the type of review, review method and health area from the lists below.

Type of review

Cost effectiveness
No

Diagnostic
No

Epidemiologic
No

Individual patient data (IPD) meta-analysis
No

Intervention
No

Living systematic review
No

Meta-analysis
No

Methodology
No

Narrative synthesis
Yes

Network meta-analysis
No

Pre-clinical
No

Prevention
No

Prognostic
No

Prospective meta-analysis (PMA)
No

Review of reviews
No

Service delivery
Yes

Synthesis of qualitative studies
Yes

Systematic review
Yes

Other
No

Health area of the review

Alcohol/substance misuse/abuse
No

Blood and immune system
No

Cancer
No

Cardiovascular
No

Care of the elderly
No

Child health
Yes

Complementary therapies

No

COVID-19
No

Crime and justice
No

Dental
No

Digestive system
No

Ear, nose and throat
No

Education
No

Endocrine and metabolic disorders
No

Eye disorders
No

General interest
No

Genetics
No

Health inequalities/health equity
Yes

Infections and infestations
No

International development
Yes

Mental health and behavioural conditions
Yes

Musculoskeletal
No

Neurological
No

Nursing
No

Obstetrics and gynaecology
No

Oral health
No

Palliative care
No

Perioperative care
No

Physiotherapy
No

Pregnancy and childbirth
Yes

Public health (including social determinants of health)
Yes

Rehabilitation
No

Respiratory disorders
No

Service delivery
No

Skin disorders
No

Social care
No

Surgery
No

Tropical Medicine
No

Urological
No

Wounds, injuries and accidents
No

Violence and abuse
No

31. Language.

Select each language individually to add it to the list below, use the bin icon to remove any added in error.
English

There is not an English language summary

32. * Country.

Select the country in which the review is being carried out. For multi-national collaborations select all the countries involved.

England

33. Other registration details.

Name any other organisation where the systematic review title or protocol is registered (e.g. Campbell, or The Joanna Briggs Institute) together with any unique identification number assigned by them. If extracted data will be stored and made available through a repository such as the Systematic Review Data Repository (SRDR), details and a link should be included here. If none, leave blank.

34. Reference and/or URL for published protocol.

If the protocol for this review is published provide details (authors, title and journal details, preferably in Vancouver format)

Add web link to the published protocol.

Or, upload your published protocol here in pdf format. Note that the upload will be publicly accessible.

Yes I give permission for this file to be made publicly available

Please note that the information required in the PROSPERO registration form must be completed in full even if access to a protocol is given.

35. Dissemination plans.

Do you intend to publish the review on completion?

Yes

Give brief details of plans for communicating review findings.?

36. Keywords.

Give words or phrases that best describe the review. Separate keywords with a semicolon or new line. Keywords help PROSPERO users find your review (keywords do not appear in the public record but are included in searches). Be as specific and precise as possible. Avoid acronyms and abbreviations unless these are in wide use.

community health workers; Maternal and child health; Health equity; Resilient community health system;

Barriers; Facilitators; Sub Saharan Africa.

37. Details of any existing review of the same topic by the same authors.

If you are registering an update of an existing review give details of the earlier versions and include a full bibliographic reference, if available.

38. * Current review status.

Update review status when the review is completed and when it is published.New registrations must be ongoing so this field is not editable for initial submission.
Please provide anticipated publication date

Review_Ongoing

39. Any additional information.

Provide any other information relevant to the registration of this review.

40. Details of final report/publication(s) or preprints if available.

Leave empty until publication details are available OR you have a link to a preprint (NOTE: this field is not editable for initial submission). List authors, title and journal details preferably in Vancouver format.

Give the link to the published review or preprint.
